# Contextualized School-Based Cognitive Behavioral Therapy (CBT) Intervention for Malaysian Secondary School Students

**DOI:** 10.3389/fpsyt.2020.565896

**Published:** 2020-12-21

**Authors:** Jo Anne Saw, Cai Lian Tam, Vanlal Thanzami, Gregory Bonn

**Affiliations:** ^1^Department of Psychiatry, Faculty of Medicine, Universiti Teknologi MARA (UiTM), Sungai Buloh Campus, Selangor, Malaysia; ^2^Department of Psychology, Jeffrey Cheah School of Medicine and Health Sciences, Monash University, Subang Jaya, Malaysia; ^3^GA21 Multidisciplinary Platform & Jeffrey Cheah School of Medicine and Health Sciences, Monash University, Subang Jaya, Malaysia; ^4^The Chicago School of Professional Psychology, Chicago, IL, United States

**Keywords:** cognitive behavioral therapy (CBT), school-based prevention intervention, adolescence, depressive symptomatology, automatic negative thoughts

## Abstract

This study investigates the effectiveness of the school-based Shine Through Any Roadblocks (STAR) CBT intervention, by a screening conducted on 634 students from eight secondary schools in Malaysia. Participants (*n* = 85) who fulfilled the eligibility criteria were assigned randomly to either the intervention group (*n* = 42) or the assessment-only waitlist control group (*n* = 43). The intervention consisted of eight group-based sessions over a period of 2 months. Sessions were 60-min each and conducted according to the STAR module. Outcome measures (depressive symptoms and automatic negative thoughts) were administered at five intervals: baseline/pre-intervention, mid-intervention, post-intervention, 1-month after intervention, and 3-months after intervention. Results showed significant and lasting lower levels of depressive symptoms and automatic negative thoughts in the intervention group, indicating that the STAR intervention could be an effective means of reducing depressive symptomatology among adolescents. Clinical implications for the Malaysian secondary school context are further discussed.

## Introduction

Adolescent mental health has become a major public health concern worldwide. Research indicates that mental illness has increased among adolescents across many countries and cultures (1–4). There is compelling evidence that mental illness is an important issue as it often progresses until adulthood (5). According to the World Health Organization (WHO), approximately 350 million individuals worldwide suffer from depression (6). Thus, reducing adolescent depression, could have major benefits for overall mental health world-wide (7–9).

Depression can manifest itself in many ways; from varying degrees of mood dysfunction to profound impairment in performing daily life tasks (10). It has also been predicted to be among the most costly illnesses worldwide by 2020 due to serious morbidity (11). Adolescent depression is a strong predictor of the frequency and severity of depression in adulthood (5, 12). It is also a strong predictor of suicide attempts (13), learning difficulties (14), identity development (15) and risky behaviors (16).

Thus, adolescents with elevated depressive symptoms are more susceptible to the development of major depressive disorder in adulthood and many other problems (16). High levels of depressive symptoms have also been recorded in Malaysia. To be specific, the prevalence of depressive symptomatology among Malaysian school adolescents aged 12–17 years old was reported to range from 7.8 to 24% (17–20). Various therapies for depression have been developed. These include structured psychological interventions such as Behavioral Activation (BA), Cognitive-Behavioral Therapy (CBT), and Interpersonal Psychotherapy (IPT) which have been recommended for adults and older adolescents by the World Health Organization (21). The National Institute for Health and Clinical Excellence (NICE) also has recommended CBT, IPT, and short-term family therapy for depressed adolescents (22).

This research is based on CBT, a psychoeducational and skill-based therapy that aims to address cognitive biases and self-defeating behaviors which contribute to depressive symptoms. Among the aforementioned varieties of psychotherapies, meta-analyses indicate that CBT has the most evidence showing its efficacy for treating depression (23–26).

Although CBT is commonly used as individual psychotherapy, it has also been effectively used in group settings (27). Thus, recently the use of group CBT has increased in community settings, such as schools, because CBT can be delivered through manualized programmes by a range of professionals in different settings (28). For example, in Puerto Rico, Rossello and Bernal (29) have successfully used group-based CBT to treat adolescents with depression. Relatedly, Gaete et al. (30) found that both adolescents and parents viewed school as an appropriate setting for mental health interventions, largely due to ease of access and convenience. Again, many researchers have stressed that early intervention in schools has great potential to reduce the burden of disease and contribute to better long-term health outcomes.

CBT is an Evidence-Based Practice (EBP) for the management of depression (31). However, needs related to different cultural contexts have led to the increased use of culturally modified or adaptations of the basic approach (32). Culturally adapted CBT modules designed for adolescents with depressive symptoms have generally shown positive results (33, 34). These adapted modules retain the core components of CBT while including modifications related to cultural sensitivity, language, and accessibility.

CBT is designed for use in clinical settings as a part of a management protocol. In Malaysia, CBT is applied as the first-line psychosocial intervention in most clinical settings, such as hospitals. However, the amount of empirical research on CBT in Malaysia is relatively scarce (35). Despite the extensive use of CBT, no manual nor any standardized step-by-step CBT module is readily available to assist in the organization of a school-based intervention for depression in Malaysia. To date there has been no intervention programme conducted in Malaysia which applied CBT in school-based programmes for adolescents in a replicable manner. Therefore, this study tested a CBT module with standardized step-by-step instructions, structured contents (e.g., themes and techniques of CBT), and a flow and structure deemed to be appropriate for use with Malaysian secondary school students.

Here we endeavored to create a culturally appropriate and linguistically sensitive intervention for Malaysian secondary school students. For this purpose, we adapted and contextualized another well-established module (29, 36). The results and implications of this project are presented below.

## Methods

### Ethics Approval

Ethics approval was obtained from the Monash University Human Research Ethics Committee (MUHREC) with project code of 1332. Approval was also gained from the Ministry of Education Malaysia.

### Study Design

This study contained two phases; a screening period referred to as Phase 1, followed by the intervention study labeled as Phase 2. A cross-sectional survey was conducted in Phase 1 to assess depressive symptomatology among the adolescents using paper-and-pencil, self-rated questionnaires. The second phase of the study emphasized on the efficacy of the school-based CBT intervention module among the adolescents who exhibited elevated depressive symptomatology in Phase 1 using a randomized experimental design, which involved both the treatment and control groups with 1:1 allocation ratio in a parallel design.

### Sampling and Sample Size

All Form 4 students in each of eight participating schools participated in the screening survey (Phase 1). This number of participants that were recruited exceeded the required sample size of 217, which was determined based on the past prevalence of 17.7% (18) using the formula by Pourhoseingholi et al. (37) for a screening survey. This was executed to assist the recruitment for Phase 2.

The sample size in Phase 2 was determined by power calculations using G^*^Power 3.1 software according to the effect size from a previous study, which used the primary outcome measure, RADS-2 in school-based CBT intervention among adolescents (14) via priori power analysis (38). With a power of 0.95 and an alpha value of 0.05, a total sample size of 74 participants was required. Thus, a sample size of 82 is adequate for the objective of the study and account for any potential attrition. Considering the low to none drop-out rate from the pilot study (39), and the estimation of the dropout rate of 10%, the total number of participants including the dropout rate amounted to 82. Therefore, the total sample size for this intervention study (*n* = 82) was adequate. In order to recruit 82 participants for the experimental design in Phase 2 as well as considering the drop-outs, incomplete surveys, and decline rate of participation, a total of 669 participants were involved in the Phase 1 screening survey. Hence, this explained the recruitment in Phase 1 which involved eight schools and a total of 669 participants.

### Participants

A total of 669 Form Four students aged 16 years old from eight secondary schools in Pahang were recruited into this study in Phase 1. As stated in the approval letter from Ministry of Education Malaysia, the research could only be conducted among students who were not in major exam years, including Form 3, Form 5, and Upper 6, which explained the selection of only Form 4 students. Hence, Form 4 students who are similar of age are recruited as the participants of this study. Out of all the 669 Form Four students, 5.23% of the students failed to participate in the screening survey, in which 1.20% of the students were not granted with permission from their guardians, while 4.03% were absent during the survey administration.

From the final total of 634 participants, 306 participants were males and 328 were females. All the participants age 16 years old, with 405 Malays, 139 Chinese, 55 Indian, and 35 Aboriginal. The overall socio-economic characteristics of the participants' parents' varied in their socioeconomic status in light of education level and monthly income. In terms of education level, most of the participants' mothers (*n* = 284, 44.80%) and fathers (*n* = 284, 44.80%) had secondary school qualification as their highest level of education. Most of the participants' mothers were housewives, while the fathers were mostly teachers, mechanics, drivers, government officers, farmers, and businessmen. A majority of the families (40.10%) earned between RM 1001 and RM 3000, while 81 participants reported no income (12.60%) in the family.

**Figure 1:**
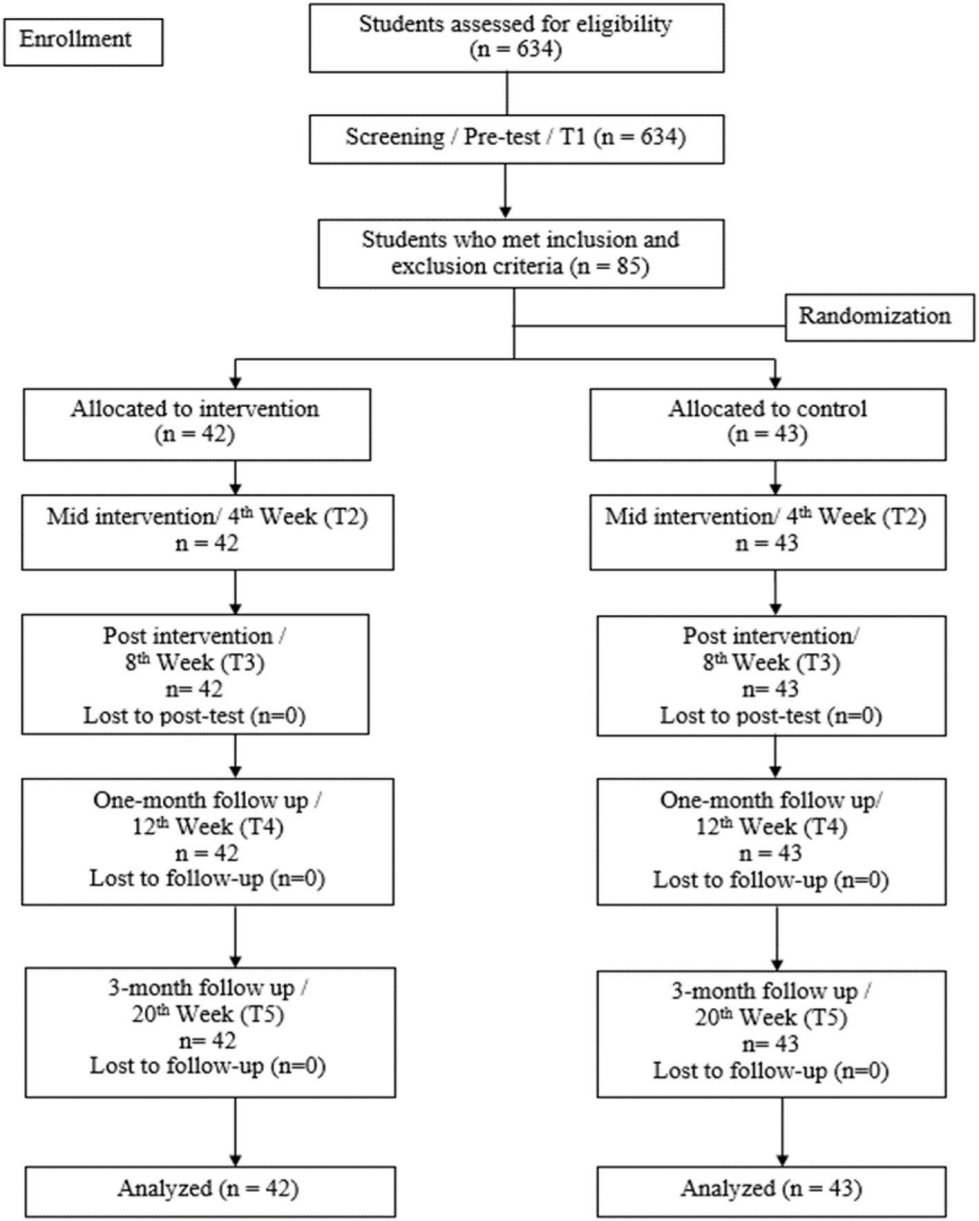
Flow diagram.

Following the screening, which was conducted on all Form 4 students in each school, the students were then chosen according to specific inclusion and exclusion criteria for the Phase 2 intervention study. The inclusion criteria consisted of [1] obtained a total raw score at and above 76 in Reynolds Adolescent Depression Scale – 2nd Edition (RADS-2); [2] competent in basic reading, including written and conversational Bahasa Malaysia; and [3] provided written consent. Meanwhile, the exclusion criteria consisted of [1] recent or previous presence of any psychiatric disorders; [2] history of neurological illness; [3] intellectually disabled; (4) recently under pharmacotherapy or psychotherapy treatment; [5] being associated with drug or alcohol abuse; and [6] indication of suicidal intent at any point of the study. The flow diagram for selection process was referred and documented in Figure 1.

During the screening process, a total of 85 participants fulfilled the criteria of the Phase 2 intervention study. The consented participants were then assigned to either CBT intervention programme group or waitlist control group. This stage was conducted by an independent research assistant, based on a stratified randomization list of different strata of depressive symptomatology, namely mild, moderate, and severe according to the RADS-2 manual (40) with three distributions (mild classification, *n* = 42, moderate classification, *n* = 19, and severe classification, *n* = 24). Stratified randomization was performed to ensure adequate representation of each subgroup of the depressive symptomatology in both the CBT intervention group and waitlist control group. In this case, simple randomization was applied using Microsoft Excel with coded numbers by the independent research assistant. The randomization was performed on the participants in each category of the symptomatology (stratified random sampling) to either the intervention group or control group. Forty-two participants were randomly assigned to the intervention group, with a distribution of 21 boys (50.00%) and 21 girls (50.00%). Specifically, the members of this group consisted of 28 Malays (66.70%), seven Chinese (16.70%), and seven Aboriginals (16.70%). The control group consisted of 43 participants, with 14 boys (32.60%) and 29 girls (67.40%), and 30 Malays (69.80%), nine Chinese (20.90%), two Indians (4.70%) and two Aboriginals (4.70%). Both intervention group and control group have participants from all the three categories of depressive symptomatology with ratio of 1:1.

### Measures

#### School-based STAR CBT Module

**Figure 2:**
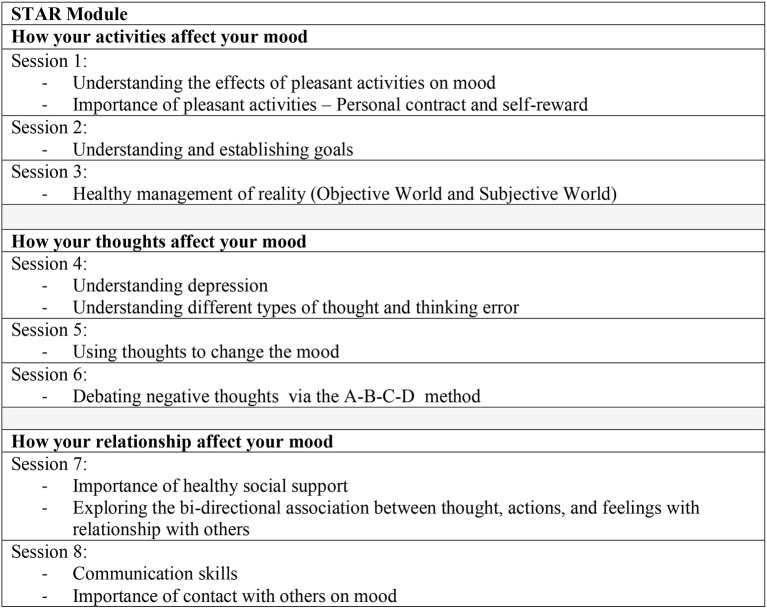
STAR module layout.

The school-based STAR CBT intervention module was the finalized version of the validated CBT module with minor amendments made based on the preliminary findings (39). Overall, this intervention module was adapted and contextualized from the “Treatment Manual for Cognitive Behavioral Therapy for Depression” by Rossello and Bernal (29). Furthermore, it encompassed the CBT core elements of thoughts, feelings, and behaviors with a focus on the psychoeducation, behavioral and cognitive changes, and improving relationships and communication skills. This module consists of eight sessions covering the three main themes in a specific sequence, namely [1] how your activities affect your mood (Sessions 1–3); [2] how your thoughts affect your mood (Sessions 4–6); and [3] how your relationship affect your mood (Sessions 7 to 8). These themes were found to be practical and helpful for adolescents in the pilot study (39). Additionally, this intervention module consists of homework, hands-on activities, and interactive discussions during the sessions which cover one session per hour on a weekly basis in a group intervention approach. Refer to Figure 2 for the layout of the module.

#### Primary Outcome Measure - Reynolds Adolescents Depression Scale – Second Edition (RADS-2)

The Reynolds Adolescent Depression Scale-2 [RADS-2, (40)] is a tool for the screening of depressive symptoms among adolescents aged 12–18 years old. It consists of 30 items, which are rated by respondents based on the four-point response format, ranging from “almost never” to “most of the time,” with higher scores suggesting higher severity. The test scores range from 30 to 120, with a cut-off score which is on par and exceeds the raw score of 76. Some of the items include “I feel sorry for myself” and “I feel life is unfair.” Notably, RADS-2 has good psychometric attributes, with an internal consistency reliability coefficient of 0.93, subscale reliability coefficient ranging from 0.85 to 0.92 (40), while a Cronbach's alpha of 0.89 was obtained in this current study.

#### Secondary Outcome Measure - Automatic Thoughts Questionnaire – Malay (ATQ-Malay)

The Automatic Thoughts Questionnaire- Malay [ATQ-Malay, (41)] is the Malay version of the original 30-item Automatic Thoughts Questionnaire (ATQ) by Hollon and Kendall (42). This questionnaire measures the occurrence of automatic negative thoughts. Furthermore, ATQ-Malay consisted of 17 negative thoughts, rated on a scale of 1 (not at all) to 5 (all the time), with higher scores indicating an increased frequency of negative thoughts. Overall, this questionnaire is a reliable measure of automatic negative thoughts, with the Cronbach's alpha coefficients ranging from 0.83 to 0.93 (41). Cronbach's alpha of 0.92 was obtained in this current study.

### Procedure

Permission to conduct this research in secondary schools was obtained from the Ministry of Education as well as the Monash University Human Research Ethics Committee (MUHREC). Following the approvals, the independent research assistant managed the recruitment list and sampling procedure. The list of secondary schools in Pahang was referred based on the online portal of the Education Department of Pahang and tabled according to the categorization by the Ministry of Education Malaysia for all secondary schools (43). A new list of secondary schools was then generated based on the pre-determined category in the research (e.g., public national secondary schools, co-educational school, and schools of urban area). This list was then randomized using the Excel software.

Based on the randomized list, school authorities were approached in sequence and the nature of the research was explained, followed by the school counselors to receive detailed information regarding the STAR CBT intervention programme. The schools' participation was highly reliant on the cooperation and willingness of the school authority although the list of schools that was generated was referred to in a sequence. Although a total of 11 secondary schools were approached to participate in the study based on the list, only 10 schools initially agreed to participate, while two schools dropped out during the screening stage, leaving eight participating schools. The decline of participation was primarily due to fear of commitment and interruption from teaching in school, lack of interest, and other different reasons including the language barrier. Administration of the screening and the intervention programmes were conducted upon the approval from the principals.

Prior to the data collection, the targeted participants (Form 4 students) were gathered after school assembly in each respective school for briefing about the research according to the Explanatory Statement, including the nature of the study, participants' rights, and confidentiality. Enquiries from the participants were addressed during the briefing. Parental consent form with an explanatory statement was distributed beforehand to the students with assistance from the counselors. The following day, the parental consent form was then collected from all the students in each school which took approximately 1 week. For the administration of the survey, participants who had obtained approval from the guardian/parents were gathered in the presence of counselors in the school hall or the respective classrooms according to the arrangement by the school authority. Another briefing was performed, with the student signing the assent form (written consent from the participants themselves). Participants were also noted that they were allowed to opt-out of participation any time during the research.

The survey booklet with demographic information, RADS-2 items, and ATQ-Malay items was distributed to the participants. The administration of the survey took about 30–45 mins to complete. A standard set of instruction was provided to ensure that all participants from all different participating schools received the same instruction and information. The survey booklets were then recollected for further analysis. A brief general report was presented to all the school principals and the counselors, and included the distribution of depressive symptomatology among the school participants. Based on the analysis, participants who fulfilled the stated inclusion and exclusion criteria for the intervention were invited to take part in the Phase 2, intervention stage. The screening with RADS-2 and ATQ-Malay among the participants was defined as time point (T1) with the score being used as the baseline score.

A briefing was made to the school counselors and participating students regarding the nature and flow of the intervention programme immediately after the completion of Phase 1. Parental consent forms were then distributed to the agreed participants, followed by the explanatory statement related to the intervention programme. Another briefing was conducted regarding the research according to the Explanatory Statement in terms of the nature of the study, participants' rights and confidentiality, and the equal chance of the participants to be placed in either the waitlist control group or the intervention group. Participants were then reminded that they should not disclose or share the materials or information that they had learnt until the completion of the research project to avoid contamination bias. Additionally, enquiries from the participants were addressed during the briefing, and the parental consent forms were recollected from the participants on the following day.

After the consent of participation in the intervention was provided by the students and their parents/guardians, the participants were assigned randomly to two different groups by an independent research assistant using the Microsoft Excel upon stratified randomization according to their depressive symptomatology classifications (mild, moderate, severe). Randomization was performed individually in each category of the depressive symptomatology (mild classification, *n* = 42, moderate classification, *n* = 19 and severe classification, *n* = 24) to either the intervention/STAR group or the control group, respectively. The participants were equally randomized in each classification with 1:1 ratio. The participants were then grouped together in small cohorts in each school according to the allocated group. The research design involved the measurement of the depressive symptomatology and automatic negative thoughts at different time points, namely pre-intervention/baseline (T1) during the screening, mid-intervention (T2/4^th^ week), post-intervention (T3/8^th^ week), 1-month follow-up (T4/12^th^ week), and at 3-month follow-up after the intervention (T5/20^th^ week).

A balanced proportion of participants were observed in both the intervention group and control group. Double blinding was unable to be executed, and hence only the participants were blinded to the treatment allocation. The student investigator who was also the only facilitator and the respective school counselors were not blinded with the treatment allocation. The eight-session school-based STAR CBT intervention programme was conducted with the intervention group in the designated counseling room during the school hours approved by the principals in each school. The intervention group participants were grouped in small cohorts of three to eight participants in each respective school based on the allocation from the stratified randomization that was performed by the independent research assistant. Logistic and administrative matters, such as the time and venue of the intervention sessions, were occasionally discussed with the respective school counselor. It was reported that no other ongoing wellness or psychological support workshop was present throughout the research procedures. Furthermore, only the student investigator was in charge of all the intervention sessions, hence standardization of trainings was assured. Each session lasted for one hour per week. All the eight sessions were conducted during different points of time of school hours every week to avoid the students from missing the lessons of the same subjects every week. Each session consisted of the basic format of homework review, weekly mood rating, display of new material, group exercises and discussion.

To facilitate the sessions, the implementation of role-playing, group presentation, games, and worksheet exercises was performed according to the manual. Participants were also assured they were free to voice out their concerns during the sessions and not obliged to share anything they were not comfortable with. Besides, as all the group members were informed of the confidentiality within the group sessions, they were reminded to not disclose the information of the sessions to other friends in schools. Additionally, light snacks were prepared for the participants during the sessions to provide a comfortable and relaxed setting. To determine whether the participants attended and observed the content of the intervention, homework and mood chart were reviewed in the next session every week. Key ideas, informal questions, and quiz session were also recapped in the following sessions, followed by another briefing on any uncertain and unsure material or content raised by participants.

Throughout the intervention until the 3-month follow-up at the school, the control group was only observed by the school counselor. Furthermore, no treatment nor related material was provided to this group aside from general counseling support by the school counselor upon participants' requests. However, no counseling session was conducted during the research period as reported by the counselors. The depressive symptomatology among this group participants was also measured by the independent research assistant at the same time with the intervention group, namely mid-intervention (T2/4^th^ week), post-intervention (T3/8^th^ week), 1-month follow-up (T4/12^th^ week), and 3-month follow-up after the intervention (T5/20^th^ week). In this case, the participants in the control group were gathered in the counseling room during each evaluation time points. The same procedure was applied to all the participants in the control group from all the participating schools during the approximate time frame. Participants in both groups could opt-out of the research at any time. Referral letter was provided when medical attention was needed, and participants were referred to the respective experts.

Continuous supervision was provided to the independent research assistant on the nature of the research and screening protocols. Being independent of the intervention sessions and blinded to the treatment allocation of the participants, the research assistant was only responsible for the assessment at the mid- and post-intervention, including its follow-ups for the intervention and control groups. The student investigator was continuously monitored and guided in terms of the execution of the sessions within the research team through weekly supervision.

The attendance of the intervention group was recorded at 90.5% during the third and fifth sessions (four absentees during each sessions), while full attendances were recorded in the remaining sessions. All participants attended a minimum of six sessions from the total eight sessions, with no dropout being recorded. Prior to each weekly session, absentees in the previous sessions were briefed about their missed session. Depressive symptoms were assessed by the independent research assistant using the questionnaire booklet, which consisted of RADS-2 and ATQ-Malay at mid- and post-intervention, including the 1-month and 3-month follow-up time points at the designated counseling room after the end of the respective session. In the case of the participants from the control group who only took part in the assessment sessions, 100% response rate was achieved as the assessment was conducted during the scheduled durations in the respective schools. During the 3-month follow-up, the participants from the control group were immediately invited into the eight-session STAR CBT intervention. However, three schools from the control group declined the 8-week involvement. Instead, the schools requested that the sessions were shortened into one brief intervention session. The participants of the three schools were also provided with printed copies of the STAR CBT module during the brief intervention session conducted at the respective schools. The other control group participants in the other five schools were involved in the complete eight-session group based STAR CBT intervention.

To conclude the research, a debriefing session was conducted in each participating school to provide closure for all the programme participants, counselors, and school authorities. In this phase, detailed information regarding the research programme, intervention materials, progress outputs, and research findings was documented. Furthermore, the participants' parents/guardians were informed beforehand regarding the debriefing, allowing the parents/guardians to share any concerns with the student investigator. However, none of the parents/guardians attended this session possibly due to work commitment during office hours. Nevertheless, no reported concern or complaint was present from the participants, parents/guardians or school authorities during the post-six-month follow-up conducted with the school counselors through an informal meeting.

## Results

This study probed into the effectiveness of the STAR school-based CBT module, with initial screening via cross-sectional questionnaire survey in Phase 1, and followed by an intervention study through experimental design that compared the intervention group with wait-list control group in Phase 2.

Table 1 shows that the distribution above cut-off score was spread across mild, moderate, and severe clinical depression cases. Based on the cut-off scores, this study yielded a prevalence rate of 13.41%.

**Table 1:** Distribution of Depressive Symptomatology (RADS-2).

	***n***	**Percentage (%)**
Normal	549	86.60
Mild clinical depression	42	6.60
Moderate clinical depression	19	3.00
Severe clinical depression	24	3.80
Total	634	100.00

Table 2 shows that the participants shared similar socio-demographic characteristics and pre-intervention scores at the baseline. Hence, no significant differences were found between the intervention and control group conditions for gender, race, religion, parents' education level, family income, number of siblings, and academic achievement. The pre-intervention scores of the outcome measures (RADS-2 and ATQ-Malay) were similar between the two groups. This portrays that all the participants were similar and comparable at the baseline level prior to the school-based STAR CBT intervention.

**Table 2:** Homogeneity in Sociodemographic Characteristics and Baseline Scores of the Participants who Participated in the Intervention Group and Control Group.

**Variables**	***t*-test**	p^*^
Gender	1.64	0.105
Race	1.07	0.288
Religion	1.43	0.158
Father's highest education level	0.914	0.364
Moher's highest education level	0.834	0.407
Family income	1.217	0.227
Number of siblings	0.433	0.666
Academic result	1.26	0.212
Pre-intervention scores (RADS-2)	0.675	0.502
Pre-intervention scores (ATQ-Malay)	0.574	0.567

* *Significant at 0.05*.

Table 3 tabulates the mean and standard deviation values of the outcome measures (RADS-2 and ATQ-Malay) for pre-intervention, mid-intervention, post-intervention, one-month follow-up after intervention, and 3-month follow-up after-intervention for both control and intervention groups.

**Table 3:** Means and Standard Deviations of Dependent Variables at Pre-Intervention, Mid-Intervention, Post-Intervention, 1-Month Follow-Up after Intervention and 3-Month Follow-Up after Intervention for Intervention and Control Group.

	**Intervention group (STAR group)**			**Control group**		
**Measure**	***n***	**M**	**SD**	***n***	**M**	**SD**
**RADS-2**						
Pre-intervention	42	83.88	4.60	43	84.37	4.68
Mid-intervention	42	81.57	4.83	43	83.88	4.97
End-intervention	42	77.14	4.85	43	81.47	5.29
Post 1-month intervention	42	74.02	4.98	43	82.74	5.25
Post 3-month intervention	42	73.17	4.99	43	81.74	5.06
**ATQ-Malay**						
Pre-intervention	42	51.79	13.64	43	50.19	12.01
Mid-intervention	42	47.62	13.36	43	52.42	11.69
End-intervention	42	45.55	10.76	43	50.40	11.47
Post 1-month intervention	42	44.26	9.42	43	49.77	10.83
Post 3-month intervention	42	43.40	9.47	43	50.79	10.00

The analysis of the effect of treatment, both via the primary and secondary outcome measures was based on intention-to-treat analysis.

### Effect of Treatment – Reynolds Adolescent Depression Scale – Second Edition (RADS-2)

The effectiveness of CBT intervention on depressive symptomatology among the adolescents was investigated using a 2 (intervention vs. control) x 5 (baseline, mid, post, 1-month follow-up and 3-month follow-up) mixed within-between repeated measures ANOVA. Treatment allocation denoted the between subject variables, whereas time points were assigned as the within-subject variables.

The assumptions of normality and homogeneity of variance were assessed using Shapiro-Wilk, F_max_, and Levene's test statistics (44). The Shapiro-Wilk statistics was not significant, indicating that the assumption of normality was not violated. Levene's Test of Equality of Error Variances was not significant at α = 0.05; *F*_(1, 83)_ = 0.01, *p* = 0.948; *F*_(1, 83)_ = 0.07, *p* = 0.800; *F*_(1, 83)_ = 0.07, *p* = 0.798; *F*_(1, 83)_ = 0.43, *p* = 0.515; and *F*_(1, 83)_ = 0.01, *p* = 0.938. The assumption of homogeneity of variance for the between-subjects factor was not violated. Homogeneity of variance assumption for the repeated measures factor was not violated with F_max_= 1.33. Therefore, no assumption was violated in the analysis.

The Mauchly's test of sphericity was significant, indicating the assumption of sphericity was violated. Thus, Huynh-Feldt Epsilon was referred. The Huynh-Feldt Epsilon was preferred over the more conservative Greenhouse-Geisser Epsilon (44).

There was a significant main effect of treatment allocation on depressive symptomatology, *F*_(1, 83)_ = 34.24, *p* < 0.001, with η_p_^2^ = 0.29.The main effect of time was also significant, *F*_(2.11, 175.26)_ = 57.98, *p* < 0.001, η_p_^2^ = 0.41 and a *post-hoc* depressive symptomatology 3-month follow-up after-intervention (M = 73.17, SD = 4.99) was significantly lower than the scores obtained for pre-intervention (M = 83.88, SD = 4.60). Refer Table 4.

**Table 4:** Main Effects and Interactions, between Intervention and Control Groups in Reynolds Adolescent Depression Scale – Second Edition (RADS-2) and Automatic Thoughts Questionnaire – Malay (ATQ-Malay).

	**STAR group (intervention group)**			**Control group**			**Treatment allocation effect**		**Time effect**		**Treatment allocation** * **Time effect**	
	***n***	**M**	**SD**	***n***	**M**	**SD**						
							***F***	***p***	***F***	***p***	***F***	***p***
RADS-2							32.24	0.000	57.98	0.000	47.69	0.000
Time 1	42	83.88	4.60	43	84.37	4.68						
Time 2	42	81.57	4.83	43	83.88	4.97						
Time 3	42	77.14	4.85	43	81.47	5.29						
Time 4	42	74.02	4.98	43	82.74	5.25						
Time 5	42	73.17	4.99	43	81.74	5.06						
ATQ-Malay							4.24	0.043	5.34	0.012	8.56	0.004
Time 1	42	51.79	13.64	43	50.19	12.01						
Time 2	42	47.62	13.36	43	52.42	11.69						
Time 3	42	45.55	10.76	43	50.40	11.47						
Time 4	42	44.26	9.42	43	49.77	10.83						
Time 5	42	43.40	9.47	43	50.79	10.00						

*Time 1, Pre-intervention; Time 2, Mid-intervention; Time 3, Post-intervention; Time 4, 1-month follow-up; Time 5, 3-month follow-up after intervention*.

In order to analyze the variances in scores between the five-time points, independently for both intervention group and control group, a paired sample *t*-test was performed. In the intervention group, the participants' RADS-2 scores exhibited a significant reduction from pre- to mid-intervention [*t*_(41)_ = 2.33, *p* = 0.025], pre- to post-intervention [*t*_(41)_ = 6.03, *p* < 0.000], pre-intervention to 1-month follow-up [*t*_(41)_ = 8.89, *p* < 0.000], pre-intervention to 3-month follow-up [*t*_(41)_ = 9.79, *p* < 0.000], mid- to post-intervention [*t*_(41)_ = 8.40, *p* < 0.000], mid-intervention to 1-month follow-up [*t*_(41)_ = 11.59, *p* < 0.000], mid-intervention to 3-month follow-up [*t*(_41)_ = 12.39, *p* < 0.000], post-intervention to 1-month follow-up [*t*_(41)_ = 9.14, *p* < 0.000], post-intervention to 3-month follow-up [*t*_(41)_ = 9.30, *p* < 0.000], and 1-month to 3-month follow-up [*t*_(41)_ = 5.29, *p* < 0.000].

As for the control group, the participants' RADS-2 scores displayed significant reduction from pre- to post-intervention [*t*_(42)_ = 2.96, *p* = 0.005], pre-intervention to 3-month follow-up [*t*_(42)_ = 2.81, *p* = 0.007], mid- to post-intervention [*t*_(42)_ = 3.59, *p* = 0.001], mid-intervention to 1-month follow-up [*t*_(42)_ = 2.13, *p* = 0.039], mid-intervention to 3-month follow-up [*t*_(42)_ = 3.44, *p* = 0.001), and 1-month follow-up to 3-month follow-up [*t*_(42)_ = 2.08, *p* = 0.044). The scores exhibited slight fluctuations between post-intervention and follow-up assessments, whereby the control group experienced significant increase in RADS-2 scores from post-intervention to 1-month follow-up [*t*_(42)_ = −2.30, *p* = 0.027], but non-significant change from post-intervention to 3-month follow-up [*t*_(42)_ = −0.95, *p* = 0.346]. Nevertheless, no significant change was noted from pre- to mid-intervention [*t*_(42)_ = 0.53, *p* = 0.599].

**Figure 3:**
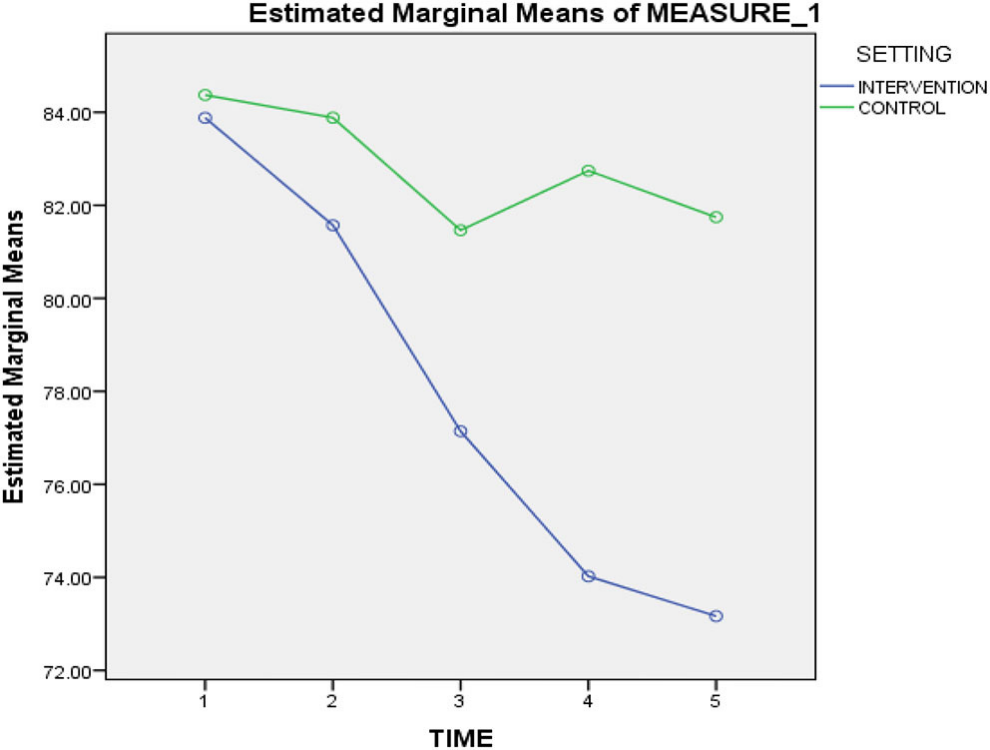
Mean scores of the RADS-2 at pre-intervention, mid-intervention, post-intervention, 1-month follow-up after intervention and 3-month follow-up after intervention.

There was a significant interaction between time and treatment [*F*_(1, 83)_ = 47.69, *p* < 0.001], with η_p_^2^ = 0.37. The variances between the groups at each assessment points were evaluated using one-way ANOVA. The pre-intervention RADS-2 scores for both intervention and control groups were not significantly different [*F*_(1, 83)_ = 0.24, *p* = 0.627], thus suggesting that the groups were similar prior to treatment. Both the intervention and control groups revealed significant changes in RADS-2 scores for mid-intervention [*F*_(1, 83)_ = 4.73, *p* = 0.032], post-intervention [*F*_(1, 83)_ = 15.41, *p* < 0.000], and 1-month follow-up intervention [*F*_(1, 83)_ = 61.65, *p* < 0.000]. Nonetheless, the intervention group portrayed significant reduction in RADS-2 scores at post-intervention. Significant differences in trend between the two groups continued until 3-month follow-up after intervention [*F*_(1, 83)_ = 61.90, *p* < 0.000] with the intervention group displaying further reduction. Figure 3 illustrates that the rate of reduction from pre-intervention to 3-month follow-up intervention was more rapid, especially for the intervention group than the control group. This highlighted that the control group retained higher RADS-2 scores throughout the varying time points until the 3-month follow-up after-intervention, compared to the intervention group that maintained lower RADS-2 scores. Refer Table 4.

As Figure 3 depicts, participation in the eight-session STAR CBT intervention corresponded, on average, with a statistically significant reduction in depressive symptoms that continued through 3-month follow-up after-intervention. The control group, on average, displayed fluctuations on depressive symptoms and remained higher in scores than those recorded for the intervention group.

### Effect of Treatment – Automatic Thoughts Questionnaire- Malay (ATQ-Malay)

Shapiro-Wilk, F_max_, and Levene's test statistics were performed to analyze the normality and the homogeneity of variance (44). The Shapiro-Wilk statistics supported the assumption of normality, whereas the assumption of homogeneity of variance for the between-subjects factor was not violated. The Levene's Test of Equality of Error Variances are not significant at α = 0.05; *F*_(1, 83)_ = 1.74, *p* = 0.190; *F*_(1, 83)_ = 0.53, *p* = 0.470; *F*_(1, 83)_ = 0.30, *p* = 0.584; *F*_(1, 83)_ = 0.70, *p* = 0.405; and *F*_(1, 83)_ = 0.92, *p* = 0.340. Homogeneity of variance assumption for the repeated measures factor was not violated as well with F_max_= 2.095. No assumption was violated in the analysis.

The Mauchly's test of sphericity was significant, which signified that the assumption of sphericity was violated. Therefore, the Huynh-Feldt Epsilon was referred. The Huynh-Feldt Epsilon was preferred over the more conservative Greenhouse-Geisser Epsilon (44).

The significant main effects for time, *F*_(1.48, 122.50)_ = 5.34, *p* = 0.012, η_p_^2^ = 0.06, and treatment allocation, *F*_(1, 83)_ = 4.24, *p* = 0.043, η_p_^2^ = 0.05 were found for ATQ-Malay scores using repeated measures of ANOVA. The paired sample *t*-test was carried out independently for both intervention and control groups to assess the variances in scores between the five various time points.

As for the STAR intervention group, no significant change was observed in ATQ-Malay scores between pre- to mid-intervention [*t*_(41)_ = 1.94, *p* = 0.060]. The ATQ-Malay scores decreased significantly from pre- to post-intervention [*t*_(41)_ = 2.96, *p* = 0.005], pre-intervention to 1-month follow-up [*t*_(41)_ = 3.44, *p* = 0.001], pre-intervention to 3-month follow-up [*t*_(41)_ = 3.81, *p* < 0.000], mid- to post-intervention [*t*_(41)_ = 2.54, *p* = 0.015], mid-intervention to 1-month follow-up [*t*_(41)_ = 3.19, *p* = 0.003], mid-intervention to 3-month follow-up [*t*_(41)_ = 3.58, *p* = 0.001], post-intervention to 1-month follow-up [*t*_(41)_ = 2.33, *p* = 0.025], post-intervention to 3-month follow-up [*t*_(41)_ = 3.58, *p* = 0.001], and 1-month follow-up to 3-month follow-up [*t*_(41)_ = 2.40, *p* = 0.021]. Therefore, significant reductions were noted over the course of time points.

The control group exhibited only significant changes from mid- to post-intervention [*t*_(42)_ = 3.57, *p* = 0.001], and mid-intervention to 1-month follow-up intervention, [*t*_(42)_ = 4.08, *p* = 0.000]. Nonetheless, no significant variance was noted between the scores at other time points.

A significant interaction between time and treatment was found [*F*_(1, 83)_ = 8.56, *p* = 0.004], with η_p_^2^ = 0.09. Further analyses using one-way ANOVAs had been performed. The results revealed that the scores on ATQ-Malay at pre-intervention [*F*_(1, 83)_ = 0.33, *p* = 0.567), and mid-intervention [F_(1, 83)_ = 3.11, *p* = 0.081] did not significantly differ between intervention and control groups. Significant variances were only noted between the two groups in post-intervention [*F*_(1, 83)_ = 4.03, *p* = 0.048], 1-month follow-up [*F*_(1, 83)_ = 6.24, *p* = 0.014], and 3-month follow-up after-intervention [*F*_(1, 83)_ = 12.21, *p* = 0.001]; with the intervention group displaying further reduction in the ATQ-Malay scores. Hence, the control group consistently reported higher ATQ-Malay scores with fluctuations, while the intervention group consistently maintained lower scores throughout the five time points. Refer Table 4.

**Figure 4:**
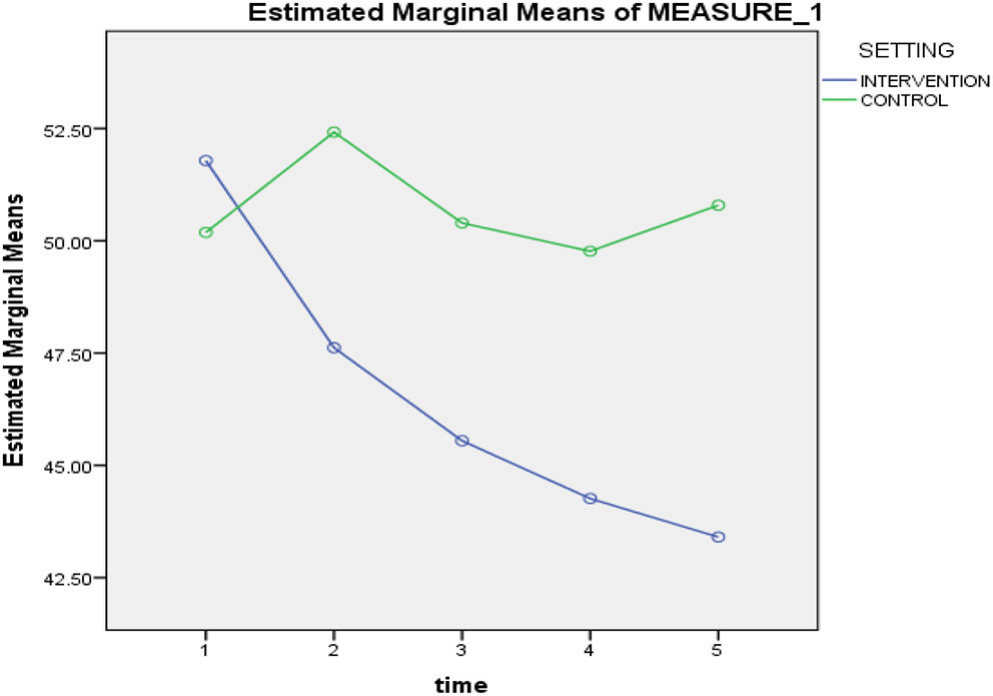
Mean scores of the ATQ-Malay at pre-intervention, mid-intervention, post-intervention, 1-month follow-up after intervention and 3-month follow-up after intervention.

Figure 4 illustrates that completion of the eight-session STAR CBT intervention corresponded to a significantly greater reduction in automatic negative thoughts, which persisted until the 3-month follow-up after-intervention, in comparison to that recorded for the control group.

Table 5 shows that the intervention group displayed significant improvements in RADS-2 scores from pre- to post-intervention, with large effect size; RADS-2 [*t*_(41)_ = 6.03, *p* = 0.000, *d* = 0.93], which was well-maintained in 3-month follow-up [*t*_(41)_ = 9.79, *p* = 0.000, *d* = 1.51]. Meanwhile, the intervention group improved from pre- to post-intervention in ATQ-Malay with small effect size [*t*_(41)_ = 2.96, *p* = 0.005, *d* = 0.46] and increased to medium effect size at 3-month follow-up [*t*_(41)_ = 3.81, *p* = 0.000, *d* = 0.59]. Treatment effects were maintained at both follow-ups (45). The control group exhibited small effect size (*d* = 0.45 and *d* = 0.43) at both post-intervention and 3-month follow-up in RADS-2, whereby the effect size for ATQ-Malay had been very small (45).

**Table 5:** Within-Treatment Effect Sizes (Cohen's d) for RADS-2 and ATQ-Malay at Post-Intervention and 3-Month Follow-Up for Intervention Group.

**Measure**	**Group**	**Post-intervention**	**3-month follow-up**
		**Effect size, *d***	**Effect size, *d***
RADS-2	Intervention	0.93	1.51
	Control	0.45	0.43
ATQ-Malay	Intervention	0.46	0.59
	Control	0.01	0.04

As portrayed in Table 6, the percentage of participants who had exerted depressive symptomatology above the cut-off point decreased across the time intervals in the intervention group. In the 3-month follow-up after-intervention, the effectiveness of CBT intervention was observed with 71.40% (30 out of 42 participants) from the intervention group which had resumed to the normal range while only 2.30% (1 out of 43 participants) from the control group had resumed to the normal range according to RADS-2 classification. Nevertheless, significant reduction was observed as none of the participants fell under the severe clinical range from post-intervention (week 8) that sustained until 3-month follow-up after-intervention in the intervention group. The stated recovery, however, was not observed in the control group.

**Table 6:** Percentage of Participants in Intervention Group and Control Group who Achieved Changes on Depressive Symptomatology based on RADS-2.

		**Time 1 (%)**	**Time 2 (%)**	**Time 3 (%)**	**Time 4 (%)**	**Time 5 (%)**
Normal range	Intervention group	0	9.50	38.10	66.70	71.40
	Control group	0	0	4.70	2.30	2.30
Mild clinical depression range	Intervention group	38.10	54.80	45.20	26.20	23.80
	Control group	60.50	46.50	55.80	41.90	53.50
Moderate clinical depression range	Intervention group	21.40	28.60	16.70	7.10	4.80
	Control group	23.20	37.20	30.20	41.80	37.20
Severe clinical depression range	Intervention group	40.50	7.10	0	0	0
	Control group	16.30	16.30	9.30	14.00	7.00

*Time 1, Pre-intervention; Time 2, Mid-intervention; Time 3, Post-intervention; Time 4, 1-month follow-up; Time 5, 3-month follow-up*.

### Teacher/Counselor Feedback on the Program

Several school counselors provided verbal feedback in a meeting with the student investigator at the end of the intervention program. One of the counselors observed some positive changes among the participants at school; be it during the regular purported individual counseling sessions, as well as during the brief discussion shared between the counselor and the participants. The counselor narrated how one participant changed her perception about her friendship issues using the cognitive skills of correcting and challenging maladaptive thoughts by applying the techniques taught during the session. Additionally, it was reported that the participant had reduced her visit to counseling sessions, as she had begun solving her own problems. The counselor stated that the participants shared how they had managed their own emotional problems with the skills acquired, while noting changes in their mood along with the request to reduce their counseling sessions in school.

## Discussion

In the current study, the effectiveness of the school-based STAR CBT intervention was assessed to reduce depressive symptomatology among adolescents through the experimental design. The cognitive changes during the intervention were also examined, which was followed by the identification of significant reliable changes in the depressive symptomatology scores among the intervention group compared to the control group.

No significant differences were found between the intervention and control groups in terms of the presentation of the elevated depressive symptomatology and automatic negative thoughts before the implementation of the school-based CBT programme, indicating that the groups were comparable, including an efficacious random assignment to the two conditions. Notably, both groups exhibited similar scores for the presentation of depressive symptomatology (RADS-2) and automatic negative thoughts (ATQ-Malay) prior to intervention (Refer Table 2). These groups were also comparable in the aspects of gender, race, religion, parents' highest education level, family income, number of siblings, and academic result, suggesting that the changes in depressive symptomatology were unlikely to be influenced by the demographic properties of the participants.

The findings suggest that the outcome indicators have answered the research question by highlighting that the school-based STAR CBT intervention could successfully manage depressive symptomatology and automatic negative thoughts among adolescents with elevated depressive symptoms. In a general overview of the trend of the mean scores, it was found that the reduction in depressive symptomatology was more significant, consistent and faster in the intervention group compared to the control group during the STAR CBT intervention.

On average (as indicated by the mean), the reduction in the participants' RADS-2 scores in the intervention group was by 2.31 points, with 95% of CI [0.30, 4.32] from pre- to mid-intervention during the fourth week, while a reduction of 6.74 points was recorded from the pre- to post-intervention in the 8th week, with 95% of CI [4.48, 8.99]. As for the pre- to one-month follow-up, a reduction of 9.86 points was obtained, with 95% of CI [7.62, 12.10], while 10.71 points reduction with 95% CI [8.50, 12.92] was achieved in the pre-intervention to 3-month follow-up. Overall, the intervention group showed a notable decrease in the RADS-2 scores from the baseline to post-intervention, which continued to decrease throughout the two follow-ups. In this case, the same inclination was observed by Merry et al. (46) in RADS scores within three follow-ups for 18 months. Upon 3-month follow-up, a significantly lower level of depressive symptomatology was identified from the adolescents in the intervention group compared to the control group. A similar trend was observed in the ATQ-Malay scores among the participants in the intervention group. On average, the participants' ATQ-Malay scores were reduced by 4.17 points, with 95% of CI [−0.18, 8.51] from pre- to mid-intervention on the fourth week, and 6.24 reduction of points were observed, with 95% of CI [1.98, 10.49] from the pre- to post-intervention on the 8th week. Moreover, 7.52 points reduction with 95% of CI [3.10, 11.95] was recorded from the pre-intervention to 1-month follow-up, while 8.38 point reduction with 95% of CI [3.94, 12.82] as recorded from the pre-intervention to 3-month follow-up. The presentation of the findings validates the objective of the study, which focused on the effectiveness of the school-based STAR CBT intervention. In this case, the intervention group consistently reported significant changes in depressive symptoms, including cognition toward the expected direction. Moreover, the strength of the changes was also evident through the large effect size observed within the intervention group. This finding was supported by the results by Mukhtar et al. (47) and Low et al. (48), who revealed that CBT was able to elicit significant changes in depressive symptoms and negative cognition.

While a certain degree of decrease was observed from the control group scores in both RADS-2 and ATQ-Malay, inconsistency and fluctuation within the time intervals were also observed. To be specific, the RADS-2 scores decreased by 0.49 points with 95% of CI [−1.37, 2.35] in the 4th week, and 2.91 points decrease was recorded with 95% of CI [0.92, 4.89] in the 8th week. However, the RADS-2 scores increased at the fourth time point during the 1-month follow-up on the 12th week by 1.63 points with 95% of CI [−0.28, 3.54], followed by 1.00 point reduction with 95% of CI [0.03, 1.97] on the 3-month follow-up. Meanwhile, ATQ-Malay scores increased by 2.23 points with 95% of CI [−7.37, 2.90] at the 4th week, and 1.02 points increase was recorded with 95% of CI [−2.42, 0.38] from 1- to 3-month follow-up after intervention. However, careful interpretation of the changes in the control group was warranted, and a reduction in the scores in RADS-2 was found until the 8th week of the research period. A possible explanation for this is the placebo effect. To illustrate, during the assessment evaluations, the control group was also gathered in the respective counseling room during each time interval (4th week, the 8th week, 1-month and 3-month) for a continuous assessment on the symptoms by the independent research assistant. Therefore, participants from the control group might display general changes in the view of the continuous evaluation.

Overall, the findings indicated that the effectiveness of STAR CBT intervention as a school-based programme in decreasing depressive symptomatology among adolescents persisted until the 3-month follow-up. Similar findings were also recorded in previous studies, which recorded a reduction in depressive symptoms in a post-one-month (49) and post 6-month follow-ups (34). Upon the completion of the school-based STAR CBT intervention and after one and 3-month post-intervention were conducted, the analysis of statistical significance recorded that 71.40% (30 participants out of the 42 participants) exhibited a normal range of depressive symptomatology as compared to only 2.30% (1 participant) from the control group.

Furthermore, the STAR intervention group obtained a large effect size (0.93) compared to the control group at post intervention, indicating that the participants who went through the intervention improved tremendously in terms of depressive symptomatology. Overall, this finding was comparable with the effect sizes established in a systematic review by Calear and Christensen (50), which ranged from 0.21 to 1.40. These findings suggest a remarkable advantage of the treatment by highlighting that school-based CBT intervention is an efficacious treatment for depressive symptomatology among Malaysian adolescents. It was also noted that the large effect size could be contributed by the experimental design when comparing with a wait-list control group. However, the decline in the self-reported measures in depressive symptomatology was in agreement with the results from past research on CBT as a school-based intervention (33, 34, 49, 51).

The underlying improvement in depressive symptomatology among the participants may be due to CBT theoretical basis and the distinct cultural consideration of the Latino culture instilled in the module. The three main themes of the CBT module focus on behavior, cognition, and relationship. Specifically, the behavior theme was introduced in Session 1–3 and focused on the importance and relationship between pleasant activities and mood, healthy management of reality, and goal establishment (29). As a result, participants would be able to learn the inter-connection of behavior and mood in a tangible manner. It could be seen that the techniques taught to the participants assisted them in increasing pleasant activities, which possibly reduced the behavioral depressive symptoms of disinterest in social and pleasant activities. Notably, a significant decrease was detected from the baseline (T1) to mid-intervention assessment (T2/4^th^ week), which indicated the effectiveness of the behavioral component. The STAR CBT intervention introduced the behavioral components of CBT in Session 1–3, which occurred before the mid-intervention assessment (T2/4^th^ week). Therefore, the effectiveness of the behavioral techniques in managing depressive symptoms especially among adolescentsis evident and supported by consistent findings with the literature (31, 33, 52).

Cognitive is the second theme of the CBT module, which particularly focuses on the identification and replacement of the habitual negative thought patterns from Session 4 to Session 6 of the STAR CBT intervention module. Although several experts consulted in this study predicted that Malaysian adolescents would be faced with challenges in these exercises, participants eventually understood the related concepts and processes. This understanding was possibly due to the suggestions by the experts to rearrange the flow of the themes within the eight sessions. Upon a detailed examination on the scores in RADS-2 in the experimental group, it was observed that the most significant score reduction occurred between mid-intervention (T2/4^th^ week) and post-intervention (T3/8^th^ week), indicating the presence of cognitive changes and symptoms improvements.

According to Tang and DeRubeis (53), therapeutic changes occurred after the fourth session in CBT, leading to a remarkable reduction in symptoms known as the “sudden gain.” Accordingly, participants elaborated on the capability of changing negative thought patterns to improve their mood, and some participants also mentioned the virtuous cycles created from this capability. To be specific, the improvement in their mood increased their tendency to be involved in pleasant activities, contributing to more positive feelings and vice versa. With this, it was indicated that cognition, behavior, and mood components influenced one another to reduce the depressive symptoms. As a result, a further decrease in depressive symptoms was observed as a continuation of the interaction between the skills taught in CBT. Overall, this pattern was closely related to the patterns reported in previous CBT research [e.g., (49)].

The selection of participants of the same age group contributed to an added advantage to the efficacy of the STAR CBT intervention. The comparable cognitive development among the participants of the same age, which was 16 years old, possibly contributed to the comprehension of the module. Provided that there was no age gap between the participants, the elements and range of discussion during the intervention possibly enhanced the understanding and absorption of the module, especially the cognitive component. Besides, a broad age range between the participants in schools was found to be less effective in teaching and learning during the CBT intervention program due to the involvement of cognitive lags (54).

The third theme of the CBT module is the relationship, which took place from Session 7 to Session 8 and guided the adolescents on the association of thoughts, actions, and feelings with the relationship with others, communication skills, and establishment of healthy social support (29). This theme was referred to as necessary by the experts, as indicated through the participants' feedback. Essentially, adolescence is the stage where the function of relationships, especially interpersonal relationships, has significant emotional impacts (55). This notion was supported by the counselor in the participating school in this study, who mentioned that one of the participants stated that she could challenge her own negative thoughts which affected her relationship with her friends. This in return, improved her mood.

Considering the “interpersonal” aspects of the Latino culture, the nature of cultural sensitivity in the module is compatible with the local context. This module focuses on specific values including *familismo* (“placing great emphasis on the family”) and *respeto* (“respect”), which were mostly reflected in the third theme (29, 56). Therefore, it was indicated that the adaptation of the interpersonal aspects into STAR CBT module was related and compatible with the Malaysian culture, which proved that it was equally important as the aforementioned values. As a result, the effectiveness of the school-based STAR CBT intervention increased, as shown in the measured outcome indicators. Notably, this finding demonstrates the effectiveness of the STAR CBT module in its objective of contributing to an efficacious CBT-type intervention among adolescents in the Malaysian cultural context [e.g., (57)].

Besides improvement, the effectiveness of the adapted intervention withstood a 1- and 3-month post-follow-up, suggesting the thriving achievement and retention of the emotional-behavioral regulation techniques. Notably, similar to the finding by Wijnhoven et al. (34), depressive symptomatology continued to decrease during the follow-ups. As highlighted by Gillham et al. (58), it took time for the participants to apply and utilize the skills learned during the intervention. The applications might occur after the intervention period when the trials and functionality of the skills became efficient in day-to-day problems or issues.

### Constraints in Transporting Evidence-Based Program Into School Settings

Challenges are among the significant aspects of this study, having to implement a school-based intervention program in schools. These challenges included the scheduling of sessions, perception of an overwhelming task, stringent protocols in schools, and the lack of appreciation of the value of the research. Similarly, these challenges were experienced in the study by Miller et al. (54) regarding school-based CBT anxiety prevention programme in Western Canada. Besides, the application of evidence-based protocol on school system requires alignment and collaboration.

Despite the suggestions made to conduct the STAR CBT programme after school-hours or at the weekends, this approach did not contribute to convenience in terms of logistics and management in school. It was also considered that [1] the school counselor was required after working hours; [2] the students had to stay back late, resulting in difficulties in going home; [3] principals had to ensure the safety of the students; and [4] the participation rate might be lower. Notably, participants also raised their concern about being left behind in a certain subjects if they missed class. Therefore, tremendous effort was required in the scheduling of sessions, in which the time and day of each week were alternated to prevent the participants from missing the same subject every week during school hours. This problem was solved by taking less influential subjects, such as Moral, Physical Education, Art, and relief classes upon the absence of the teacher-in-charge. Subsequently, although the workload of the respective school counselors would be increased with this arrangement, the school counselors became highly motivated and cooperative in this research study due to their understanding of the importance of the intervention programme to the students.

There was a lack of appreciation among certain school authorities regarding the importance of the programme due to the inadequate understanding of the value of the research in the long-run. The intervention programme was considered non-academic, while the immediate constraints and additional workload were the only aspects considered by the school authorities. Furthermore, some parents and the students themselves instilled negative mindsets on mental health and intervention, which could be explained by poor knowledge, low exposure to mental health, or the stigma around mental health (32). For these reasons, some of the students chose not to participate in the intervention programme, while some failed to obtain consent from parents. All these challenges emphasized that awareness and acceptance to psychological wellness are highly important in this era.

Nevertheless, these challenges strengthened the main objective of this research, which is to implement the STAR CBT intervention programme in Malaysian secondary schools as an annual programme. With an official implementation, this programme could be a formal programme with its time, slots, and priority being incorporated into the academic calendar. As a result, this action would solve the logistic, management, workload, and the aforementioned scheduling problems. However, as proposed by Sawyer et al. (59), the development of clear goals between the intervention programme and the school authorities are crucial for effective engagement between the teachers and students. Additionally, sufficient time should also be provided for the application of this programme in schools.

### Strength of the Study

The positive consideration implemented in this research is a notable aspect of this study due to its contribution to the findings. Some methodological considerations from past studies were addressed in this research. As highlighted by Grimes and Schulz (60), it is important to ensure the internal validity of a research from being dampened by selection bias, information bias, and confounding factor.

The generation and randomization of the condition allocations among participants to the intervention and control groups were performed on the adolescence level. The participating schools exhibited comparable demographic properties, such as urban area, public school, same category of school type, and size. These randomization within the comparable demographic properties further supported the effectiveness of the STAR CBT programme as the intervention and control groups could be compared without selection bias. This study in general, eliminates possible selection bias, accidental bias, and fulfilled the basis for the statistical analysis (61).

While the randomization within each school invited biases, the concern of contamination bias was addressed. A briefing was conducted to the participants regarding the nature of the research and the condition allocations. Although each participant was provided with the equal opportunity to be placed in either the intervention or control groups through simple randomization, the participants were advised during the first briefing to not share or discuss with others regarding any of the materials, assessments, and techniques learned throughout the intervention processes up to the 3-month follow-up assessment. This concern was also voiced out in a written form in the explanatory statement.

Several methodological considerations were addressed to reduce the biases mentioned in past studies. To be specific, the experimenter effect was reduced as the continuous assessments of the depressive symptoms through the survey booklet were conducted by an independent research assistant at four-time intervals. Similarly, to minimize the information bias, the independent research assistant was blinded to the allocation between the intervention and control groups (60).

### Limitations and Future Recommendations

Besides the strength of the research, several limitations were also present, which suggested a new direction for future studies. Firstly, this research was limited to the reliance solely on two self-report outcome measures, which might affect the measured outcome. Although the questionnaires were designed specifically to measure depressive symptoms and negative thoughts (40, 41), it is recommended for future studies to take additional outcome measures into account to determine more broad aspects of behavioral, emotional, and cognitive concerns of the adolescents, including their quality of life. Furthermore, multiple methods of reporting are also recommended to enhance the research validity (62). Additionally, the integration of this method with multiple rater approach (e.g., the counselor's evaluation) could reduce self-reporting bias in the symptoms. However, it is equally important to carefully balance the need for measurement with the time, cost, and available school staffs.

The generalization of results is another limitation. To illustrate, the generalization of these study results to all the secondary school students in Malaysia could not be performed as the data collected from the students was limited to one state only. Besides, the selection of participants was limited to Pahang, which might result in selection and sampling bias. As a result, the interpretation of the results could not be generalized to students outside Pahang as it would threaten the external validity. Provided that this research served as an initial baseline platform, the replication of findings across larger and broader samples is recommended for future research.

Putting the justification and initial intention of the possible primer research for school-based CBT for depressive symptoms, and the best fit methodological consideration, careful interpretations of the findings in reference to Malaysian context are recommended. Moreover, the initial implementation of school-based CBT led to various challenges in implementing a more rigorous research design at the current stage. Provided that the programme was new and the resources were lacking, the procedures and outcomes of the research would clearly be impacted, which would warrant attention in future research.

The significance and large effect size of the intervention may be partly explained by the incorporation of the waitlist control. However, the comparison arm using the waitlist control was acceptable for the evaluation of novel intervention or research within the context, as indicated in Mohr et al. (63). Although this approach was reviewed as modest, it was perceived in this study as potentially useful as a baseline study while addressing the arising challenges. Subsequently, stronger study design could be implemented, with the incorporation of other parallel arms (active or attention control) groups. This approach can be made to gain more comparative measurement of the effectiveness of any intervention although it would lead to an expected reduction in terms of effect sizes.

Moreover, the investigation of different potential factors, such as cultural values or religiosity insights, is also recommended as it could possibly mediate the effects of the treatment, especially in a rich multicultural society like Malaysia (48, 64). In addition, although promising results were achieved from the 3-month follow-up assessment in terms of depressive symptomatology and negative thoughts, the long-term effect of the intervention on depressive symptomatology among the adolescents was not measured in this study. Therefore, it is recommended that this aspect is investigated in the future.

### Research Implications

It is hoped that the implementation of the school-based STAR CBT intervention programmes could be performed on a large scale in all secondary schools around Malaysia. The achievement of this target would allow Malaysian youths to develop the necessary skills to be happier and healthier throughout their lives. The findings of this research have contributed to potential implications on the early prevention and intervention of depressive symptomatology among adolescents. With the promising findings from this research, improved results of school-based treatment on depressive symptoms among the adolescents could be achieved. Furthermore, the effective reduction in the presentation of the elevated depressive symptoms among the target group would also indirectly reduce the risk of adulthood depression (16) and other social and interpersonal issues (65–67). The behavioral and cognitive techniques taught in the school-based STAR CBT would eventually benefit the adolescents in a wider context, which does not only encompass their personal growth and self-regulation. It would also cover the social context, which includes their family members, friends, and teachers (68).

There was a potential for the school-based STAR CBT programme to be extended to more secondary schools and adolescents who were exposed to the risk of elevated depressive symptoms in Malaysia. This finding was supported as the extension of the STAR CBT intervention programme to the school counselor was possible with a manualized module, and it could be compatible with the school-based modality. It is recommended that the implementation of the school-based CBT in future research to be extended to the school counselor as the counselor is the prime figure in the school, who takes on the role of the well-being screener to the adolescents in schools.

In respect of the training of the counselors, previous research had demonstrated the possibilities and effectiveness of training non-mental health professionals to conduct CBT in schools (69, 70). This finding was also supported by the constraints of manpower and experts in the field of mental health inside and outside of clinical settings in Malaysia (17). Moreover, provided that previous research had demonstrated the possibilities and effectiveness of training non-mental health professionals to conduct CBT in schools, interactive training for the school counselors to deliver CBT programmes in school should be explored (69, 70) to ensure sustainability of the programme. With the promising outcomes of the intervention and being cost-effective, the training of school counselors will be beneficial for the students and schools.

Overall, this research has provided further insights into the growing body of research regarding depressive symptomatology and school-based CBT intervention in Malaysia for adolescents. As a result, the gap of knowledge regarding the methodological concern in research would be filled, and the evidence-based research study could be adopted into the community setting with a manualized module to be used and referred by the school counselors in Malaysia.

## Conclusion

This study created a contextualized and validated CBT module to be used among Malaysian adolescents who experience clinical depressive symptomatology. This approach is one of the essential steps to transport an evidence-based intervention to the Malaysian school setting. Referring to Rossello and Bernal's (29) treatment module for adolescents, the validation and incorporation of the feedback from expert reviewers and careful procedures to produce an eight-session school-based Bahasa Malaysia treatment module (STAR) were performed (39). As a result, a potential for the implementation of the module in Malaysian secondary schools could be seen.

Depressive symptoms were shown through the screening to be common among adolescents, as indicated in previous Malaysian studies (17–20). Therefore, the prevalence of depressive symptomatology in Malaysia is comparable to its prevalence in other countries according to the current global trend. However, due to the fear that depression might be a troubling and more alarming health concern for adolescents in the future, it was predicted that schools were among the first spots for early detection and intervention programs. As schools have the closest proximity to the adolescents (71), school-based intervention could serve as a good platform for adolescents. Furthermore, the concept of creating awareness about mental health ideally originates from school to prevent the stigma, which creates barriers for psychological help (72).

Based on the findings, the practicality and feasibility of the intervention as an annual programme in secondary schools in Malaysia holds a significance despite the challenges in this study. With the experimental study, the promising effects of the intervention could be illustrated in further studies, which explore the use of the school-based STAR CBT intervention in more Malaysian school settings. Furthermore, this study has filled in the knowledge gap from multiple perspectives through methodological consideration and clinical implications of school-based CBT for adolescents with depressive symptomatology, as highlighted in the existing literature. The findings shed light on the effectiveness and importance of early intervention. Therefore, school-based STAR CBT intervention may be a good alternative in terms of the cost and benefit of the programme.

## Data Availability Statement

The raw data supporting the conclusions of this article will be made available by the authors, without undue reservation.

## Ethics Statement

The studies involving human participants were reviewed and approved by Monash University Human Research Ethics Committee (MUHREC). Written informed consent to participate in this study was provided by the participants' legal guardian/next of kin.

## Author Contributions

JAS was the primary author in presenting the write-up. CLT, GB, and VT contributed in data collection, design, and analysis. JAS and CLT were involved in the conceptualization and analysis of results. All authors contributed to the article and approved the submitted version.

## Conflict of Interest

The authors declare that the research was conducted in the absence of any commercial or financial relationships that could be construed as a potential conflict of interest.
